# *Low-dose naltrexone* plays antineoplastic role in cervical cancer progression through suppressing PI3K/AKT/mTOR pathway

**DOI:** 10.1016/j.tranon.2021.101028

**Published:** 2021-02-01

**Authors:** Ning Liu, Limei Yan, Fengping Shan, Xiaonai Wang, Na Qu, Mike K Handley, Mingxing Ma

**Affiliations:** a Department of Obstetrics and Gynecology, Shengjing Hospital of China Medical University, No. 36 Sanhao Street, Heping District, Shenyang 110004, Liaoning, China; b Department of Immunology, College of Basic Medical Science, China Medical University, No. 77 Puhe Road, Shenyang North New Area, Shenyang 110122, Liaoning, China; c Department of Gynecology, Cancer hospital of China Medical University, Liaoning Cancer Hospital & Institute, No. 44, Xiaoheyan Road, Dadong District, Shenyang 110042, Liaoning, China; d Cytocom Inc., 37 North Orange Avenue, Suite 607, Orlando, FL 32801, USA; e Department of General Surgery, Shengjing Hospital of China Medical University, No. 36 Sanhao Street, Heping District, Shenyang 110004, Liaoning, China

**Keywords:** Low-dose naltrexone, Cervical cancer, Proliferation, Invasion, PI3K/AKT/mTOR, OGF, opioid growth factor, LDN, low-dose naltrexone, PI3K, phosphatidylinositol 3 kinase, EMT, epithelial-mesenchymal transition, NSCLC, non-small cell lung carcinoma, VEGFR, vascular endothelial growth factor, EGF, epidermal growth factor, HGF, hepatocyte growth factor, FGF, fibroblast growth factor

## Abstract

The incidence of cervical cancer is increasing annually worldwide. Low-dose naltrexone (LDN) has been reported to delay tumor progression, but the mechanism remains unclear. Here, we found that low-dose naltrexone could upregulate the expression of OGFr. Additionally, LDN could suppress the abilities of colony formation, migration and invasion in cervical cancer cells. LDN could also inhibit cervical cancer progression in mice model. Moreover, LDN indirectly reduced the expressions of PI3K, pAKT and mTOR in vitro and in vivo. Therefore, LDN may be considered a potential treatment option for cervical cancer.

## Highlights

• LDN inhibited proliferation in cervical cancer.
• LDN inhibited migration and invasion in cervical cancer cells.
• LDN mediated the propagation property in cervical cancer through PI3K/AKT/mTOR signaling pathway.

## Introduction

Approximately 570 000 cases of cervical cancer and 311 000 deaths from the disease occurred in 2018 [1]. Among females, cervical cancer was the fourth most common cancer, ranking after breast cancer, colorectal cancer and lung cancer. Cervical cancer was the leading cause of cancer-related death in women in eastern, western, middle, and southern Africa. China and India together contributed more than a third of the global cervical burden, with 106 000 cases and 48 000 deaths in China [2]. Human papillomavirus (HPV) infection is the main cause of cervical cancer has resulted in the development of prophylactic vaccines to prevent HPV infection and HPV assays that detect nucleic acids of the virus. With the improvement in diagnostic technology and medical treatment, the outcome of patients with cervical cancer has significantly improved; however, the prognosis of patients with regional and distant metastasis is still poor. Unfortunately, a significant number of patients with cervical cancer who undergo a supposedly curative operation develop local recurrence or distant metastasis leading to shorter survival. Thus, preventing metastasis is fundamental to treat cervical cancer.

Many viruses are associated with the development of this tumor and that there isone reason for chronic infection, inflammation and the role of cytokines ininflammation and carcinogenesis [3], [4]. Accumulating evidence suggests that the phosphatidylinositol-3-kinase/protein kinase B (PI3K/Akt) signaling pathway is vital for the growth, metabolism, apoptosis, metastasis, chemotherapy resistance of cancer cells [5], [6]. PI3K/Akt signaling pathway has been demonstrated to mediate EMT process [7], [8]. Furthermore, the activation of the PI3K/Akt/HIF-1α signaling pathway has been found to play a pivotal role in mediating hypoxia-induced EMT transformation and invasion in rheumatoid arthritis-fibroblast-like synoviocytes (RA-FLSs) [9] and hypoxia-induced EMT and chemoresistance in hepatocellular carcinoma [10]. In lung adenocarcinoma, Mex3a interacts with LAMA2 to promote metastasis via PI3K/AKT pathway [11].

The opioid growth factor (OGF) and its receptor, OGFr, play a regulatory role in cell proliferation, and maintain homeostasis through a tonically active negative feedback mechanism. OGF is an inhibitory growth factor that is autocrine and paracrine produced and targets normal and abnormal replicating tissues [12], [13]. The OGF-OGFr axis can be blocked by opioid antagonists such as naloxone and naltrexone. Naltrexone was developed in 1963 as an orally active opioid receptor antagonist to be used in the treatment of opioid and alcohol addiction. It is FDA-approved for the indication of medical-assisted treatment of alcoholism or opioid use disorder, and is prescribed in doses of 50–100 mg daily. Low-dose naltrexone (LDN) in the 1–5 mg per day range seems to work beyond the opioid receptor antagonism and modulate neuro-inflammatory processes involving inflammatory cells such glial cells. It is reported that a paradoxical response of analgesia and anti-inflammatory action at low doses [14]. Recently, more and more studies reported that Naltrexone was initially found to have functions of tumor inhibition and immunomodulation when used at low doses [15]. Naltrexone has a strong blocking effect on OGFr [16]. Zagon et al. reported that LDN could compete with OGF, after a short period of inhibition, and promote the increase of OGF. Low-dose naltrexone inhibits colorectal cancer progression and promotes apoptosis by increasing M1-type macrophages and activating the Bax/Bcl-2/caspase-3/PARP pathway [17].

In the present study, we found that OGFr was low expressed in tumors of patients with cervical cancer. Low-dose naltrexone could upregulate the expression of OGFr. Additionally, LDN could suppress the abilities of colony formation, migration and invasion. LDN could also inhibit cervical cancer progression in mice model. Moreover, LDN indirectly reduced the expressions of PI3K, pAKT and mTOR in vitro and in vivo. Therefore, LDN may be considered a potential treatment option for cervical cancer.

## Materials and methods

### Cell culture

The human cervical cancer cell lines Hela and Siha (TCHu187 and TCHu113, Cell Bank of the Chinese Academy of Sciences) were cultured in DMEM and MEM (Gibco, USA) with 10% fetal bovine serum, 2% penicillin and streptomycin at 37 °C under 5% CO_2_. The siRNA- OGFr sequence: 5’-CCCTGGACTACTTCATGTT-3.

### Western blot and antibodies

SDS loading buffer. Samples were boiled for 15 min at 95 ° Western blot analysis was performed as described previously [18]. Proteinsamples were mixed with 4× SDS loading buffer.Samples were boiled for 15 min at 95 °C, and proteins were separated by SDS page and transferred to PVDF membranes,and then, the following primary antibodies and dilutions were used: OGFr antibody(1:1000, Sigma, USA), VEGFR2 and p-VEGFR2 antibody (1:1000, CST, USA),PI3K and PDK1 antibody (1:1000, Santa Cruz, USA), AKT, pAKT antibody, and β-actin antibody (1:1000, Proteintech, China), and mTOR antibody (1:1000, Wanleibio,China). Secondary antibodies were anti-rabbit or anti-mouse HRP conjugated IgG(1:10000, beyotime, China).

### Formation of stable cell clones

Hela and Siha cells at a density of 200 per group were seeded into 35 mm Petri dishes and cultured for 24 h. For treatment with LDN, medium containing 1.24 (Hela) or 1.83 (Siha) mg/mL LDN (Amquar Bio Co., Ltd, USA) was added. The medium were changed every 3 days for all groups. Visible colonies were observed after approximately 14 days. Then, the colonies formed were fixed with 4% formaldehyde for 20 min, followed by visualization treatment with 0.3% crystal violet staining for 5–8 min. After washing twice with PBS, the clones were scanned, photographed, and counted. Colony formation rate = (number of colonies/numbers of seeded cells) × 100%.

### Scratch assay

Scratch assays were performed as described previously [19], [20]. Before the scratch assay, the medium of each cell group treated with LDN was replaced with a serum-free medium and cultured for 1 h. The wound scratch was created using a 200 μl pipette tip on the cell surface of the Petri dish, which was then washed once with serum-free medium. The cells were observed under a microscope and photographs of each treatment group were captured. The locations of the cells in the photographs were recorded for comparison with more photographs at later check points. The cells were continually incubated in serum-free media for another 24 or 48 h before more photograph recordings at corresponding time points. The distance of cell migration in each experimental group was calculated.

### Invasion assay

Invasion assays were performed as described previously [21]. Boyden chambers were coated with Matrigel (BD Biosciences, USA). According to the manufacturer's protocol, cells (3 × 10^4^) were seeded on Matrigel in the upper chamber, and the bottom chamber was filled with 30% FBS culture medium as chemoattractant. Cells that invaded through the Matrigel-coated membrane after 24 h were fixed with paraformaldehyde and stained with 0.4% crystal violet. The fold change in invasion was calculated by dividing the number of cells in LDN treated cells by the number of cells in the control cells.

### Quantitative real-time PCR

Total RNA from tissue samples was isolated with TRIzol (Invitrogen, USA). qPCR was performed in the CFX96 Real-Time PCR Detection System (Bio-Rad, USA) using TransStart Top Green qPCR SuperMix (Transgene, China) according to the manufacturer's instructions. The primers used for PCR amplification were as follows: PIK3CA forward, 5′- CCACGACCATCATCAGGTGAA -3′ and reverse, 5′-CCTCACGGAGGCATTCTAAAGT-3′; PDK1 forward, 5′- CTGTGATACGGA TCAGAAACCG -3′ and reverse, 5′-T TCCACCAAACAATAAAGAGTGCT -3′; AKT1 forward, 5′- TCCTCCTCAAGAATGATGGCA′ and reverse, 5′- GTGCGTTCGATGACAGTGGT -3′; mTOR forward, 5′- TCCGAGAGATGAGTCA AGAGG -3′ and reverse, 5′- CACCTTCCACTCCTATGAGGC -3′; and VEGFR2 forward, 5′- GTGATCGGAAATGACACTGGAG -3′ and reverse, 5′- CATGTTGGTCACTAACAGAAGCA -3′and β-actin forward, 5′- CATGTACG TTGCTATCCAGGC-3′and reverse, 5′- CTCCTTAATGTCACGCACGAT-3′. qPCR data were analyzed using the comparative Ct method, and the expression of target genes was normalized to that of β-actin.

### Tumor cell implantation in nude mice

BALB/C nude mice (4–6 weeks old) were purchased from Charles River (Beijing, China). The Ethics Committee of the China Medical University approved the experiments. A total of 4 × 10^6^ Hela cells (in 0.1 mL solution) were injected into nude mice at the right flank via subcutaneous injections (five mice/group). The mice were randomly divided into four groups, including the control, the concentrations of LDN 0.5 mg/kg, 5 mg/kg, and 10 mg/kg. Intraperitoneal injection of naltrexone dissolved in physiological saline with corresponding concentration began the day after tumor cell implantation. Tumor growth and body weights were measured every 3 days, and the tumor volume was calculated with the formula: (length × width2)/2 (mm^3^). Thirty-six days after the tumor was implanted, retro-orbital blood collection was performed before euthanizing the mice to collect and weigh the tumors.

### Statistical analysis

All statistical analyses were performed using GraphPad Prism Software. The results are expressed as mean values ± SD. Each assay was performed in at least three independent replicates. Significant differences between two groups were assessed using paired two-tailed Student's *t*-tests. A *p*-value of ≤0.05 was considered statistically significant. *p* ≤0.05, ≤0.01, ≤0.001 and≤0.0001 are represented by single, double, triple and four asterisks respectively.

## Result

### Examining the expressions of OGFr in patients tumors with cervical cancer

**Fig. 1:**
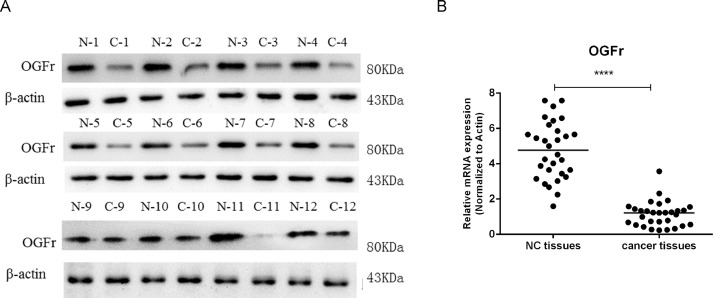
OGFr was low expressed in cervical cancer tumors. (A&B) The expressions of OGFr in patients’ tumors with cervical cancer were examined by Western Bolt and qPCR. N was Normal tissues and C was cancer tissues. Data represent the mean±SD.

To check the expression of OGFr in patients’ tumor with cervical cancer, western blot was performed in 12 patients. We found that OGFr was low expressed in cervical cancer tissues (Fig. 1A). Furthermore, we also checked the expression of OGFr in mRNA level of tissues, quantitative PCR (qPCR) was performed in 60 specimens: 30 specimens of cervical cancer tissues and 30 of paired adjacent non-cancerous tissues. We found that OGFr was significantly downregulated in cervical cancer tissues (Fig. 1B).

### LDN suppresses proliferation, migration and invasion in cervical cancer cells in vitro

**Fig. 2:**
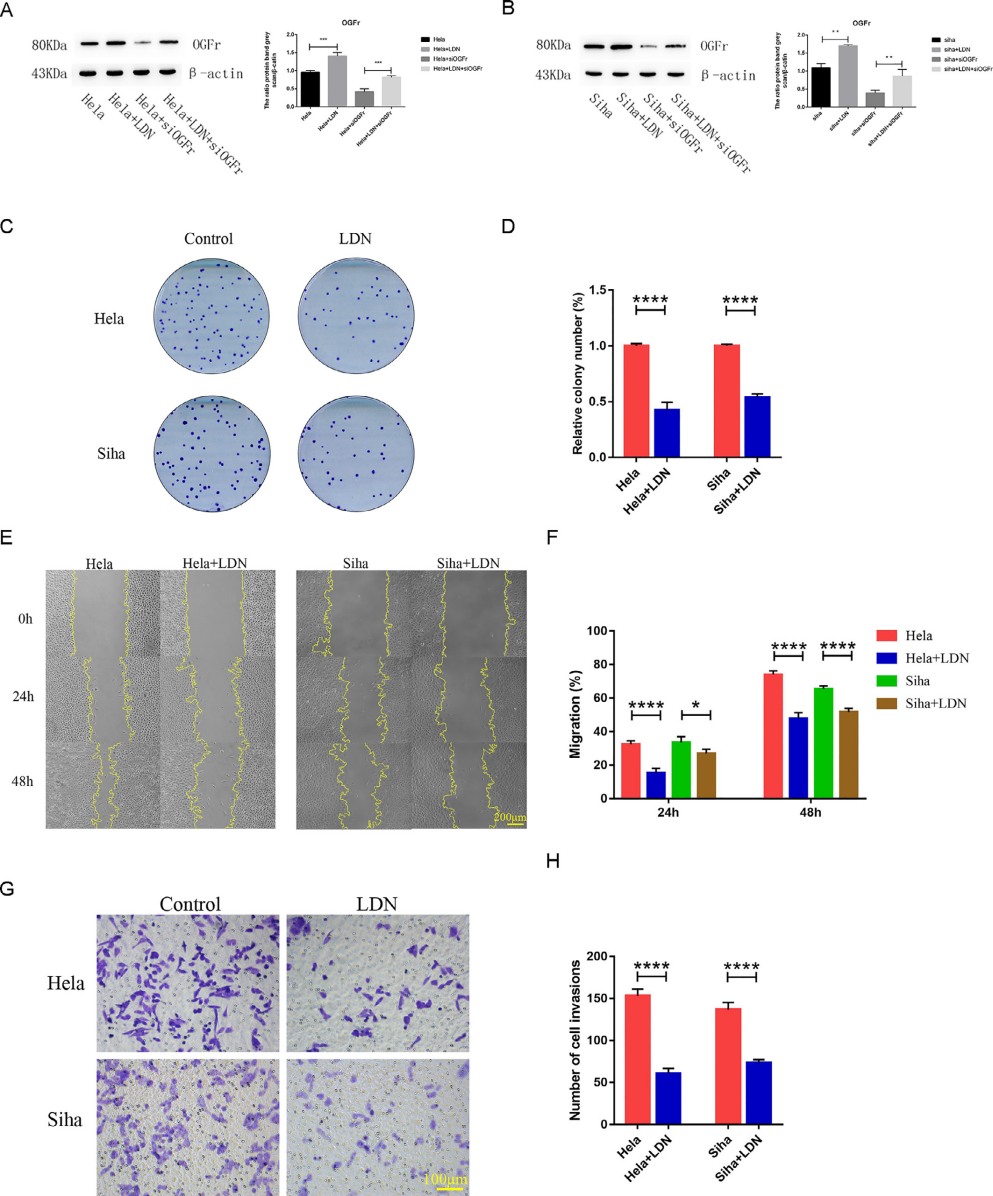
LDN inhibited proliferation, migration and invasion in cervical cancer cells. (A&B) The expressions of OGFr in cervical cancer cells treated LDN were examined by Western Bolt. (C&D) The influence of LND treated on colony-formation ability in Hela and Siha cells. (E&F) The migration of Hela and Siha cells were analyzed by Scratch assay. The statistical analysis was shown in the bar graph (mean ± SD from three independent experiments), and a representative experiment was shown. (G&H) The invasiveness of Hela and Siha cells were analyzed in invasion assay. The fold change in invasion was shown in the bar graph (mean ± SD from three independent experiments), and a representative experiment was shown. For treatment with LDN, medium containing 1.24 (Hela) or 1.83 (Siha) mg/mL LDN was added.

Naltrexone has a strong blocking effect on OGFr, and we detected the expression of OGFr when cervical cancer cells treated with LDN. We found that treatment with LDN increased the OGFr expression in Hela and Siha (Fig. 2A&B). When knockdownthe expression of OGFr, treated with LDN could also increase the OGFr expression in Hela and Siha (Fig. 2A&B). To explore the function of LDN in cervical cancer cells, we detected the abilities of proliferation, migration and invasion when cervical cancer cells treated with LDN. Then the effect of LDN on cell holoclone formation was first assessed by clonogenic assays. The results showed that LDN could inhibit the holoclone formation of Hela and Siha cells (Fig. 2C&D). The scratch assay demonstrated that LDN treatment could markedly reduce the migration of Hela and Siha cells (Fig. 2E&F). Similarity, the effect of LDN on the invasiveness in cervical cancer cell lines, the results indicated that the number of invasive cells was significantly reduced (Fig. 2G&H).

### LDN mediate the propagation property in vivo

**Fig. 3:**
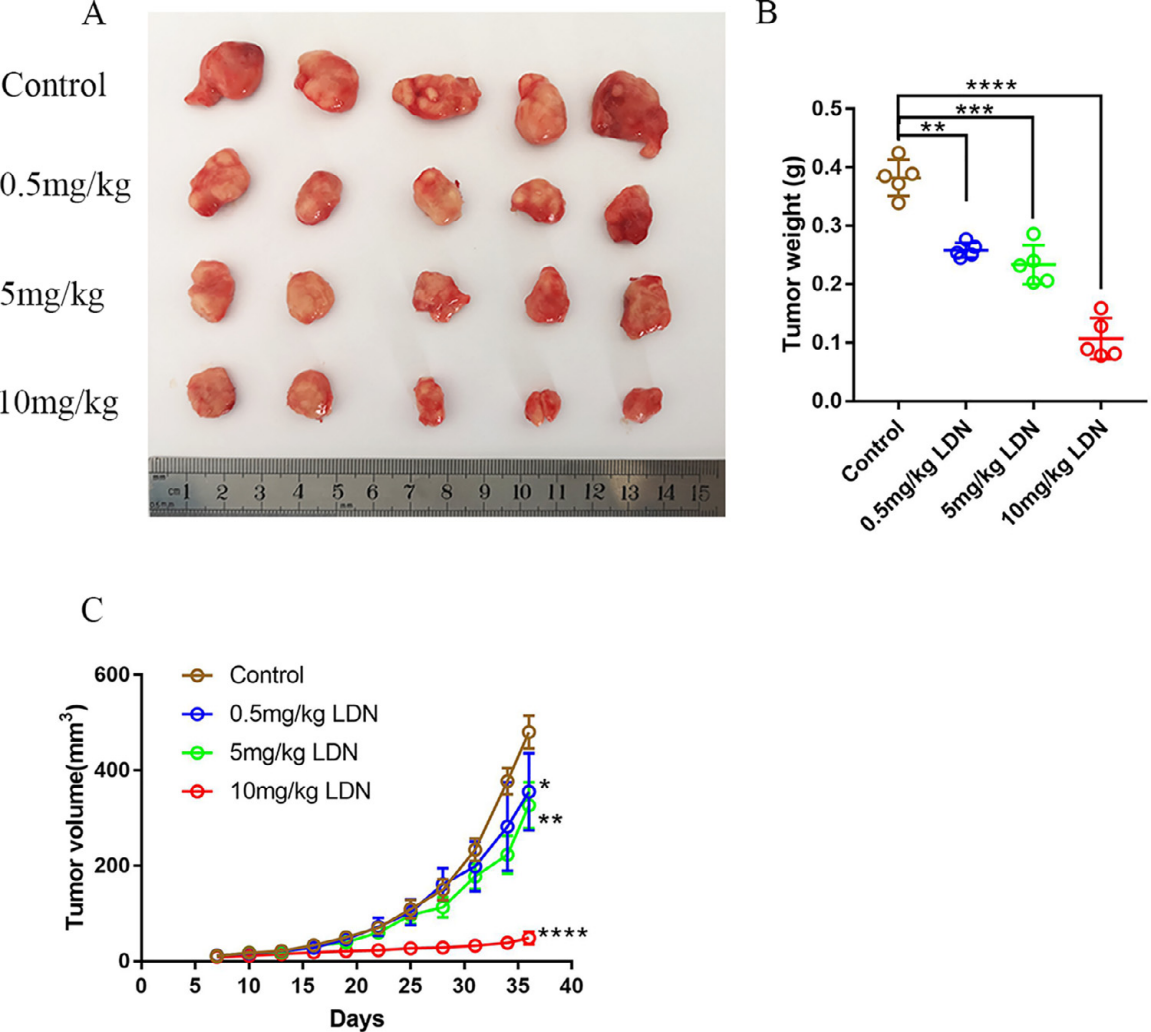
LDN inhibited the progression of subcutaneous xenografts in vivo. (A) 4 × 10^6^ Hela cells (in 0.1 ml solution) were injected into BABL/c nude mice. The nude mice experiments were performed in four independent groups. Different dose of LDN were intraperitoneally injected as 0.5 mg/kg, 5 mg/kg, 10 mg/kg. The control group of nude mice received an equal volume of normal saline. (B) Tumor weigh were determined after nude mice death. (C) Tumor volume was determined each three days. *N*=5 biologically independent samples.

Furthermore, we extended our findings to a xenograft model in vivo, in which cervical cancer cells are subcutaneously injected into mice. We noticed that LDN could inhibit tumor growth (Fig. 3A&B), as compared to control group. In addition, compared with the control group, the 10 mg/kg LDN treatment group had significant differences in inhibition on tumor growth from the 22 days of the treatment, while the 5 mg/kg LDN-treated group was 31 days. The time of significant difference in mice treated with 0.5 mg/kg LDN was 34 days (Fig. 3C). Therefore, the results showed that LDN could suppress the tumor growth with dose dependent in vivo.

### LDN mediate the propagation property in cervical cancer cells through PI3K/AKT/mTOR signaling pathway

**Fig. 4:**
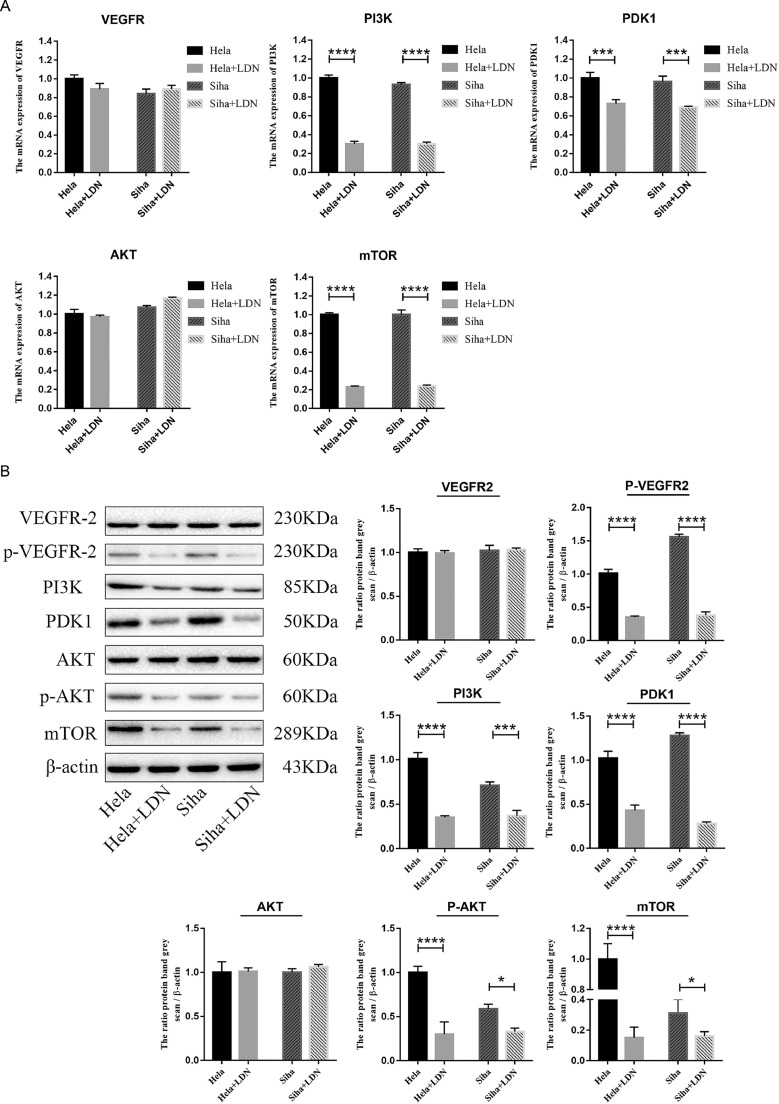
LDN mediated the propagation property in cervical cancer cells through PI3K/AKT/mTOR signaling pathway. (A&B) Changes of PI3K/AKT/mTOR signaling pathway-related protein in Hela and Siha cells treated LDN were examined by qPCR and Western Blot. For treatment with LDN, medium containing 1.24 (Hela) or 1.83 (Siha) mg/mL LDN was added.

**Fig. 5:**
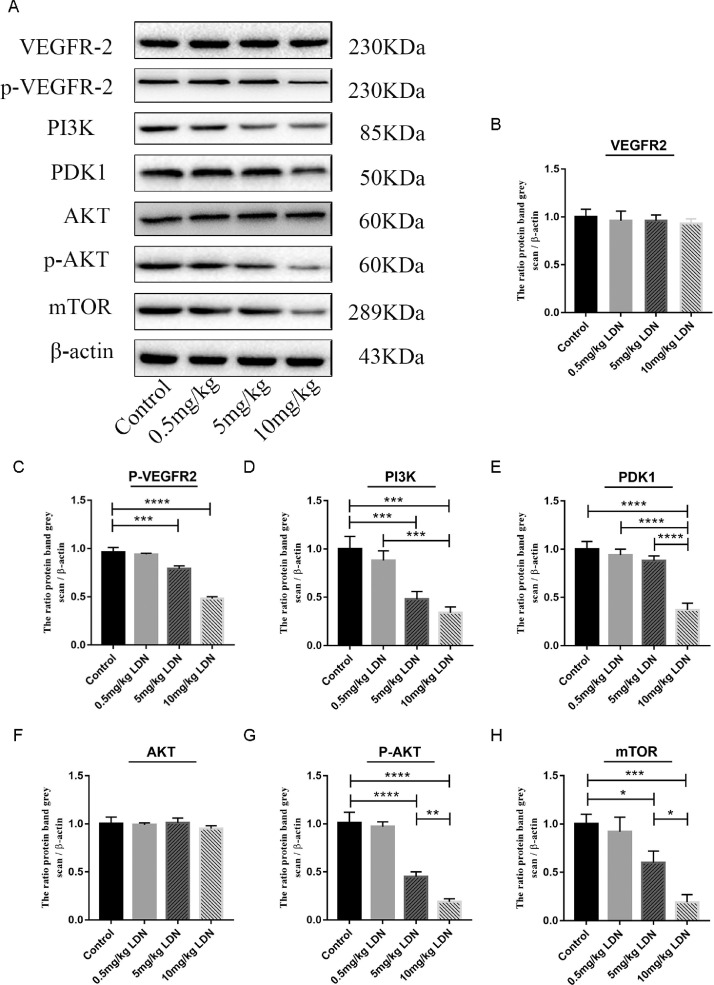
LDN mediated the propagation property through PI3K/AKT/mTOR signaling pathway in vivo. (A–H) Changes of PI3K/AKT/mTOR signaling pathway-related protein in tumors of mice treated LDN were examined by Western Blot.

PI3K/AKT/mTOR signaling pathway was involved in oncoprotein-mediated upregulation of EMT-related transcription factors in NSCLC cells. To explore whether the LDN effect was mediated the PI3K/AKT/mTOR signaling pathway, we detected the expression of VEGFR, PI3K, PDK1, AKT and mTOR by qPCR and western. As shown in Fig. 4A, LDN significantly decreased the expression of PI3K, PDK1 and mTOR. Although, there was no difference in the expression of VEGF and AKT, the expression of pVEGFR2 and p AKT was downregulated when treated with LDN in cervical cancer cells (Fig. 4B). We also found that the expression of pVEGFR, PI3K, PDK1, pAKT and mTOR significantly reduced after LDN treatment, especially in the 10 mg/kg group (Fig. 5).

## Discussion

Naltrexone, is a kind of non-specific opioid receptor antagonist, including the classical opioid receptors can adversely affect opioid receptors (μ-, κ-, δ- receptor) and the non-classical ζ-receptor (opioid growth factor receptor, OGFr) [22]. Studies have shown that opioid growth factor (OGF) interacts with OGFr to regulate the occurrence and development of tumors [23]. LDN can block the interaction of OGF-OGFr. In this study, we found that OGFr was low expressed in tumors of patients with cervical cancer. Low-dose naltrexone could upregulate the expression of OGFr.

Low dose naltrexone (LDN) gradually play a role of treatment in the wider fields, such as cancer, immune related diseases, complications related to diabetes [24], [25] mental disorders, even also AIDS [26], However, the mechanism of the role of LDN in these disease remains unclear. It was reported that LDN could inhibit non-opioid receptor Toll-like receptor 4 (TLR4) on macrophages such as microglia cells to reduce inflammatory response, and further alleviate pain symptoms caused by inflammation [27]. In colorectal cancer, LDN could inhibit CRC progression andpromotes apoptosis. LDN could induce apoptosis of CRC cells by activating Bax/Bcl-2/caspase-3/PARP pathway [28]. In our previous study, we found that LDN can up-regulate the expression of CD83, CD80, CD40 and other molecules on the surface of bone marrow derived dendritic cells (BMDCs) in vitro, further promote the differentiation and maturation of BMDCs, promote the release of IL-12 and TNF-α, and indirectly kill tumor cells [29]. In this study, we also found that LDN could suppress the abilities of colony formation, migration and invasion. LDN could also inhibit cervical cancer progression in mice model.

The PI3K/AKT/mTOR signaling pathway regulates the basic functions of cells, such as proliferation, migration, survival and angiogenesis, and also plays an important role in the response to hypoxia and energy consumption. Activation of this signaling pathway is associated with the development of cancer [30]. When the upstream oncogene expression is amplified or mutated, the PI3K/AKT/mTOR signaling axis is also activated. The upstream genes include epidermal growth factor (EGF), vascular endothelial growth factor (VEGF), hepatocyte growth factor (HGF), and fibroblast growth factor (FGF). At the same time, this signaling pathway can also be regulated by tumor suppressor genes such as PTEN.PI3K/AKT/mTOR contributes to tumor growth, angiogenesis, metastasis and drug resistance in many cell lines and mouse xenograft models [31]. Therefore, inhibition of this pathway plays an important role in anti-tumor therapy. The inhibitors of PI3K/AKT/mTOR signaling pathway, such as sirolimus and everolimus, are also under experimental research. But a variety of inhibitor drugs have dose-limiting toxicity, including hyperglycemia, macular papules, gastrointestinal intolerance (anorexia, nausea, vomiting, dyspepsia, diarrhea) and stomatitis. A few also have serious side effects, such as severe bacterial infections and immune-mediated organ damage, including colitis, hepatitis and pneumonia, leading to the cessation of clinical trials of some inhibitors shortly after development [31]. But LDN minor side effects only include drowsiness, dizziness, vomiting and diarrhea. And in people undergoing chemotherapy, LDN adjuvant therapy can reduce the side effects caused by chemotherapy, such as LDN alleviates bone marrow suppression induced by carboplatin chemotherapy in breast cancer and kidney damage. LDN can also have a synergistic effect with cisplatin in ovarian cancer, jointly inhibiting the DNA replication and angiogenesis of tumor cells. In our study, LDN indirectly reduced the expressions of PI3K, pAKT and mTOR in vitro and in vivo. Therefore, LDN can be used as an adjuvant of clinical anti-tumor drugs in inhibiting the PI3K/AKT/mTOR signaling pathway, with high safety and low price.

## Declaration of Competing Interest

The authors declare that they have no known competing financial interests or personal relationships that could have appeared to influence the work reported in this paper.

## Appendix Supplementary materials

Image, application 1
