# Supplementary material for: Low-dose naltrexone plays antineoplastic role in cervical cancer progression through suppressing PI3K/AKT/mTOR pathway
Source: Transl Oncol. 2021 Feb 1;14(4):101028. doi: 10.1016/j.tranon.2021.101028 (PMC7859308; doi:10.1016/j.tranon.2021.101028)
Supplement: Supplementary file 1 [file mmc1.docx]

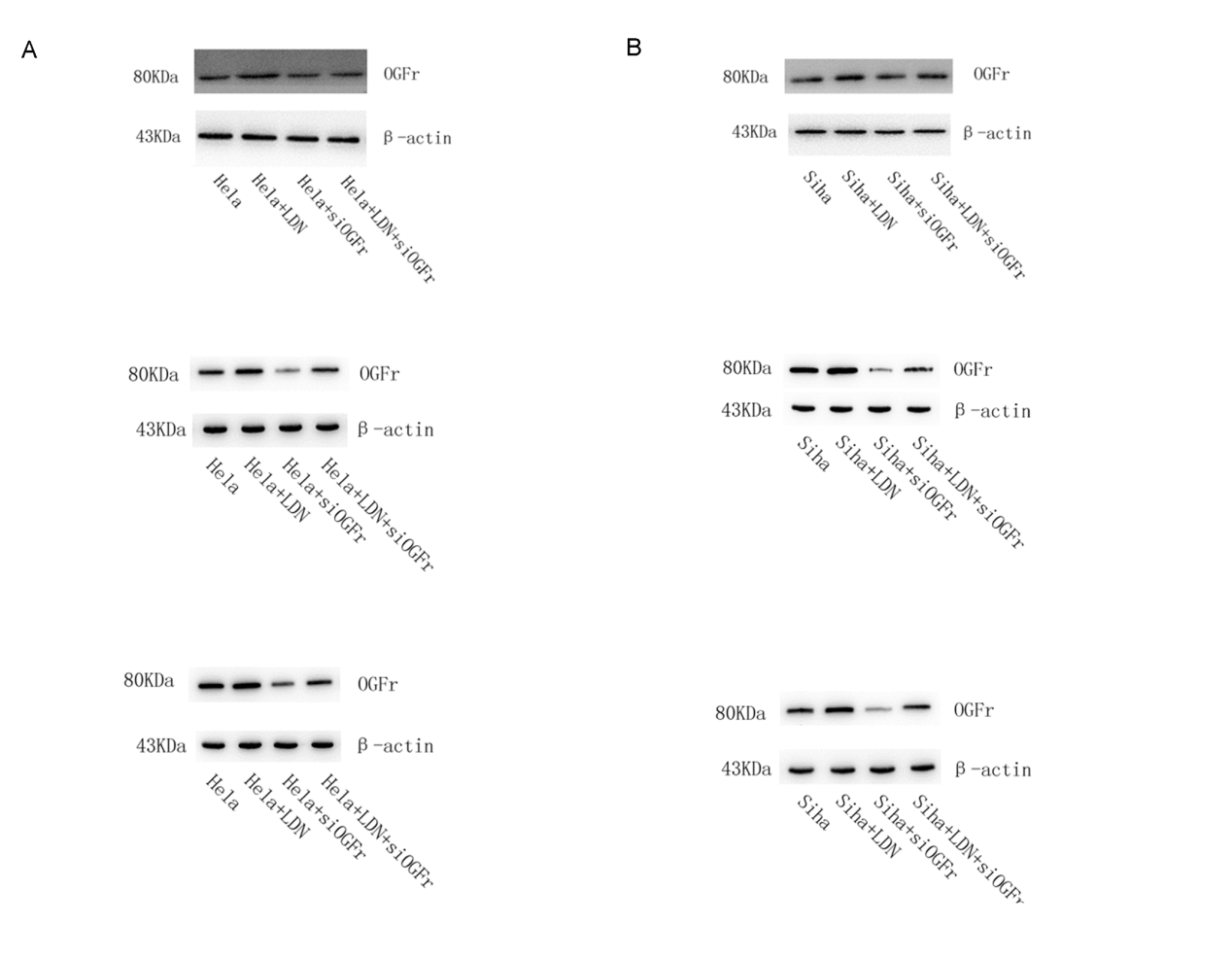


Supplementary Fig.1 (A&B) The expressions of OGFr in cervical cancer cells treated LDN were examined by Western Blot.


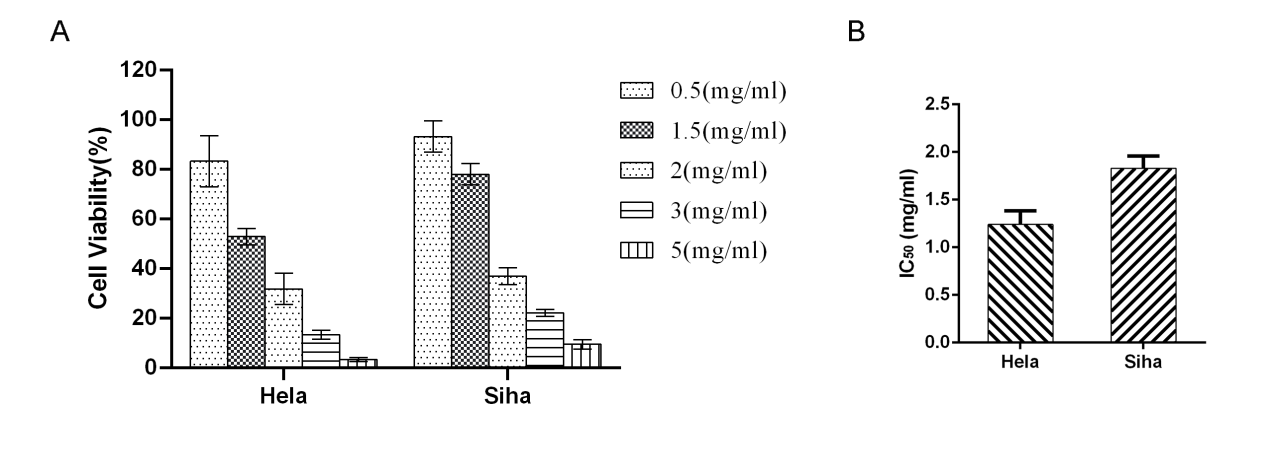


Supplementary Fig.2 Cell proliferation of LDN to Hela cells and Siha cells measured by CCK-8 assay (A) and calculated IC50 value (B)
